# Temporal Changes on the Risks and Complications of Posttransplantion Diabetes Mellitus Following Cardiac Transplantation

**DOI:** 10.1155/2018/9205083

**Published:** 2018-11-08

**Authors:** Nadia Iannino, Amine Nasri, Agnès Räkel, Anique Ducharme, Kim Lachance, Normand Racine, Simon de Denus, Maxime Tremblay-Gravel, Annik Fortier, Michel White

**Affiliations:** ^1^Montreal Heart Institute, Université de Montréal, Montreal, QC, Canada; ^2^Centre Hospitalier de l'Université de Montréal, Montreal, QC, Canada; ^3^Sanofi Canada, Laval, QC, Canada; ^4^Faculty of Pharmacy, Université de Montréal, Montreal, QC, Canada; ^5^Montreal Health Innovations Coordinating Center, Montreal, QC, Canada

## Abstract

**Background:**

Recent changes in the demographic of cardiac donors and recipients have modulated the rate and risk, associated with posttransplant diabetes mellitus (PTDM). We investigated the secular trends of the risk of PTDM at 1 year and 3 years after transplantation over 30 years and explored its effect on major outcomes.

**Methods:**

Three hundred and three nondiabetic patients were followed for a minimum of 36 months, after a first cardiac transplantation performed between 1983 and 2011. Based on the year of their transplantation, the patients were divided into 3 eras: (1983-1992 [era 1], 1993-2002 [era 2], and 2003-2011 [era 3]).

**Results:**

In eras 1, 2, and 3, the proportions of patients with PTDM at 1 versus 3 years were 23% versus 39%, 21% versus 26%, and 33% versus 38%, respectively. Independent risk factors predicting PTDM at one year were recipient's age, duration of cold ischemic time, treatment with furosemide, and tacrolimus. There was a trend for overall survival being worse for patients with PTDM in comparison to patients without PTDM (*p *= 0.08). Patients with PTDM exhibited a significantly higher rate of renal failure over a median follow-up of 10 years (*p *= 0.03).

**Conclusion:**

The development of PTDM following cardiac transplantation approaches 40% at 3 years and has not significantly changed over thirty years. The presence of PTDM is weakly associated with an increased mortality and is significantly associated with a worsening in renal function long-term following cardiac transplantation.

## 1. Introduction

Posttransplantation diabetes mellitus (PTDM), formerly called new-onset diabetes after transplant (NODAT), refers to the development of diabetes in previously nondiabetic patients, excluding transient hyperglycemia [1, 2]. Historically, the incidence of PTDM following solid organ transplantation has been difficult to determine because of the use of different diagnostic criteria. In 2002, Montori et al. [3] systematically reviewed the incidence of new-onset diabetes after heart, liver, and kidney transplantation in adults and reported 12-month cumulative incidence within the range of 2% to 53%. Similarly, Heisel et al. [4], in a systematic review, reported that the incidence of PTDM ranged from 7% to 26% in cardiac transplant (CTx) recipients, showing the large variability of incidence between the earlier studies.

International Consensus Guidelines proposed that the American Diabetes Association (ADA) criteria, published the same year for the diagnosis of diabetes mellitus in the general population, should also be applied to the organ transplant recipients [2, 5]. Nevertheless, the incidence of PTDM has remained quite variable in the previous reports, most likely because of different immunosuppressive regiments from one study to another, the evolution of immunosuppression protocols, and changes in donor and recipient characteristics over time [6]. Following kidney transplantation, the risk factors for PTDM are well established and include both general factors such as an increase in recipient age, the presence of obesity, African and Hispanic ethnicity, family history of diabetes, prediabetes prior to transplantation, as well as some transplant-specific factors such as immunosuppressive regimens including glucocorticoids, and the use of calcineurin inhibitors and/or mammalian target of rapamycin inhibitors [7–10]. In these patients, PTDM impaired long-term graft function and survival, reduced long-term overall survival, and increased the risk of mortality and morbidity associated with cardiovascular disease [11, 12]. Despite these observations in the renal transplant population, the clinical parameters associated with PTDM have been incompletely investigated following CTx [11–14]. The temporal changes assessed over a very long follow-up period have been restricted to data from the International Society of Heart and Lung Transplantation registry [15]. However, these data are limited by the lack of information regarding the donor-recipient characteristics and the specific parameters related to morbidity and mortality in the subgroup of patients who developed PTDM.

The primary objective of this study was to assess the incidence and secular trends for the development of PTDM in a large cohort of patients transplanted in one single center over 30 years. The secondary objectives were to investigate the recipient and donor characteristics over that period to determine the predictors of PTDM and its role in overall mortality and the development of renal failure post-CTx.

## 2. Methods

### 2.1. Study Design

The design of this investigation was a retrospective observational investigation and the study was approved by the Montreal Heart Institute Scientific and Ethics Committees. Data includes all patients who received a heart transplant at the Montreal Heart Institute between January 1983 and December 2010. The last follow-up date was July 31, 2013. The patients were divided into three cohorts according to the date of transplant (1983-1992 = era 1, 1993-2002 = era 2, and 2003-2011 = era 3). These eras were selected in order to subdivide this 30-year follow-up in ten-year periods. All patients who received a first transplantation and who survived hospital discharge following procedure were included in the analysis. The absence or presence of diabetes, documented at the time of surgery, was assessed by medical history and fasting blood glucose to differentiate diabetes pretransplantation from the nondiabetic patients. Patients with diabetes before transplantation were excluded from the posttransplant analyses reported in this study. The diagnosis of posttransplant diabetes was based on the initiation of hypoglycemic drugs including insulin, a fasting blood glucose ≥7 mmol/L, and/or HBA1C ≥6.5% at least once within 12 and 36 months following discharge from CTx (respectively, for PTDM 1 year and PTDM 3 years). We also assessed the rate of PTDM up to 10 year postdischarge for the whole cohort regardless of the year of transplantation. Clinical and paraclinical parameters were collected before heart transplant, at the time of hospital discharge and at each outpatient visit in the transplant clinic for at least 3 years. Outpatient visits were performed at least 1, 2, and 3 months following the intervention, every 3 months thereafter within the first year, and at least twice a year subsequently for up to 30 years. The last follow-up date was July 30, 2013. The rate of PTDM was determined at 1 and 3 years. The first occurrence of any major outcomes including all-cause death and worsening renal function defined by a decline in glomerular filtration rate below 30 ml/min/1.73m^2^ was censored during the follow-up period.

### 2.2. Statistical Analyses

Continuous variables are presented as mean ± standard deviation or median [lower and upper quartile] and group comparisons were done using Student's t-test, one-way ANOVA, Mann-Whitney-Wilcoxon test, or Kruskal-Wallis test according to the distribution of the selected variable. Categorical variables are presented as frequency (percentage) and group comparisons were performed using Chi-square test. Statistical significance was set at* p *< 0.05.

Survival to onset of PTDM was illustrated using Kaplan-Meier curves and the log-rank test was used to compare survival between eras. Univariate and multivariate Cox proportional hazard models were used to seek potential predictors for the development of PTDM in the first year after discharge. The candidate variables considered were pre-, peri-, and postoperative information collected until discharge. Variables showing a* p *< 0.20 in univariate Cox analysis were introduced in a multivariable Cox model and a stepwise selection process was used to select the final independent predictors. Cox analyses were also performed to assess the role of PTDM as a time-dependent variable in the occurrence of death or renal failure during the follow-up.

Statistical analyses were performed using SAS 9.4 (Cary, NC, USA), and statistical significance was set to* p *< 0.05.

## 3. Results

### 3.1. Study Population

**Figure 1:**
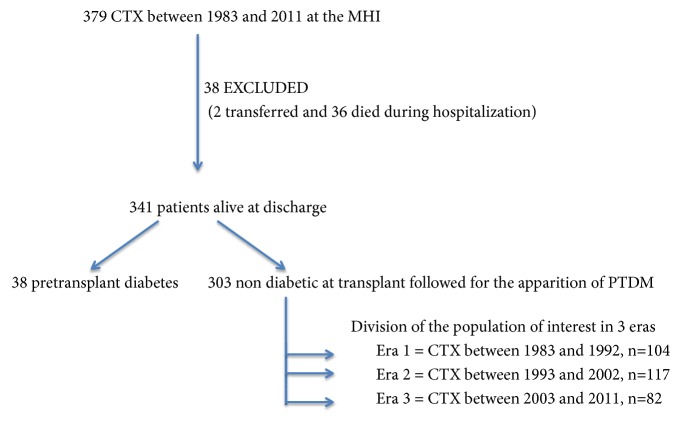
Flowchart of the study and division era.

Three hundred seventy-nine adult patients with end-stage heart failure underwent CTx at our institution between 1983 and 2011. Among these patients, forty-two patients (11%) were diabetic before transplantation and thirty-two patients died in-hospital, while two were transferred early to another hospital for their long-term care and were lost in follow-up. Consequently, our study population consisted of 303 nondiabetic patients prior to transplantation discharged alive after surgery (Figure 1). The cohort of transplanted patients was subdivided in 3 eras: era 1 (1983 to 1992) included 104 patients (34%), era 2 (1993 to 2002) included 117 patients (39%), and era 3 (2003 to 2011) included 82 patients (27%). The median duration of follow-up was 10.6 (5.3-16.4) years. The characteristics of the study population are presented in Table 1. Mean recipient age at the time of transplantation was 47±12 years. Recipient age was different between the 6 groups (3 eras, diabetic versus nondiabetics). Patients transplanted more recently (era 3) were mostly men (68%), had nonischemic heart failure etiology, and were most likely to receive inotropic support before CTx (12.5% in era 1 versus 24% in era 3;* p* = 0.07). The proportion of patients with dyslipidemia at the time of pretransplant evaluation increased with time (27% in era 1 versus 48% in era 3) while mean cholesterol and triglyceride levels decreased in era 3 (3.8 and 1.1 mmol/L) compared with era 1 (5 and 1.4 mmol/L). There were no changes in cold ischemic time between the 3 eras. The rate of cyclosporine use decreased from 100% to 30%, while tacrolimus utilization increased from 0% to 67% from 1983 to 2011. There was a significant increase in donor age and donor weight in the most recent transplant recipients. In fact, donor age increased from 27±9 years (era 1) to 37±15 years (era 3) (*p* < 0.0001) while donor weight increased from 67±13 kg to 76±17 kg in era 1 and 3, respectively (*p *= 0.0005). Similarly, donor to recipient weight ratio increased significantly in era 3 compared with era 1 (1.10±0.25 [era 3] versus 0.96±0.2 [era 1]; *p* = 0.0006). There was no difference in recipient BMI or recipient gender over the three eras. Glomerular filtration rate was also similar over the three eras (62±18 [era 1] versus 66±23 ml/kg/1.73 m^2^ [era 3];* p *= NS). More than 95% of patients were chronically treated with prednisone. The mean daily dose of prednisone was 9.79±5.06 mg per day at 6 months and 8.51±5.46 mg per day at one year. Ninety-seven percent of patients with PTDM were chronically treated with steroids compared with 100% of patients without PTDM at 1 year (*p *= 0.01).

### 3.2. Prevalence of PTDM at One and Three Years

**Figure 2:**
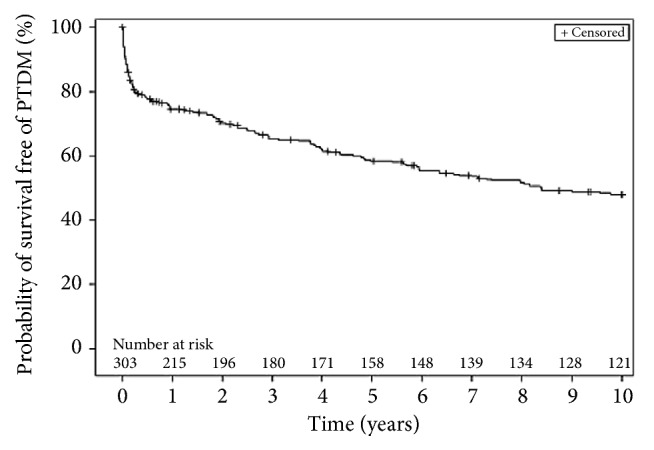
Kaplan Meyer curve for survival free of diabetes up to 10 years following cardiac transplantation.

**Figure 3:**
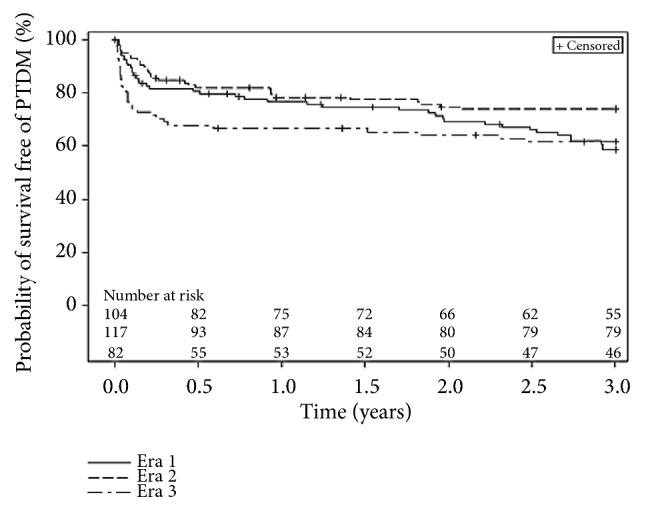
Kaplan Meyer curves for survival free of diabetes according to the transplant era. Overall* p* value; 1 year = 0.067; 3 years = 0.056.

The rates of PTDM for the whole cohort and for the 3 specific eras are presented in Table 1 and Figures 2 and 3. Seventy-six patients (25%) developed PTDM within the first year of follow-up and from those, 12 died or were lost in follow-up. Consequently, 215 CTx recipients were diabetes-free at 1 year. Cumulative survival free of diabetes was 58% at 5 years and only 48% at 10 years (Figure 2).  Figure 3 presents the Kaplan-Meier curves for the survival free of PTDM up to three 3 years after transplantation according to the era of surgery. Despite an overall borderline significance when the three eras where analyzed using log-rank test, (*p *= 0.067 and* p *= 0.056, respectively, at 1 and 3 years), subsequent intergroup analyses yielded a significantly higher rate of diabetes at 1 and 3 years in era 2 when compared with the most recently transplanted patient (era 3) (both* p *< 0.05).

### 3.3. Risk Factors and Outcome Analyses

The relationship between the risk of developing PTDM and some selected clinical and paraclinical parameters is presented in Table 2. Using univariate Cox analyses, donor and recipient age, a higher body mass index (BMI), increased blood glucose level before transplantation, a longer cold ischemic time, and the use of tacrolimus were significantly associated with an increased risk of developing PTDM at 1 year. Using multivariate Cox analyses older recipient age, longer cold ischemic time, and the use of furosemide and tacrolimus immunoprophylaxis were independently associated with an increased risk of developing PTDM at 1 year. The use of tacrolimus was the strongest predictor of PTDM with a hazard ratio close to 4-fold compared with cyclosporine immunoprophylaxis. There was a significant interaction between triglycerides level and the use of tacrolimus for the risk of developing PTDM (*p *= 0.03). Patients who developed PTDM between 1 and 3 years exhibited similar rate of use and prednisone daily dose compared with those who did not develop PTDM (not shown).

We further explored the association between PTDM, all-cause death, and the development of renal failure in our study population using the Cox proportional hazards model (Table 3). The development of PTDM yielded a* p* value = 0.08 on survival using univariate analysis and this variable was forced in the model while other parameters including age at transplantation, year of transplantation, donor gender, hypertension, use of inotrope, MPA, use of furosemide or of any other diuretics, and use of inotrope were included in a stepwise fashion in the multivariate model. Age at the time of surgery and the nonutilization of MPA were significantly associated with death, while having a female donor was protective for all-cause death. The presence of PTDM was associated with the highest risk for developing renal failure during the follow-up period (Table 3).

## 4. Discussion

In this study, we report a rate of PTDM at 1 and 3 years of 25% and 34%, respectively, in a large cohort of cardiac transplant recipients. Despite an overall statistical trend, the rate of PTDM was higher in era 3, compared to era 2, at 1 and 3 years. The development of PTDM was associated with a significant decrease in renal function over time.

The incidence and the rate of diabetes reported here are in agreement with those reported in the heart transplantation literature. Nieuwenhuis [16], Martinez [13], and Depczynski [12] reported a rate of PTDM of 19.6%, 20.3%, and 15.7%, respectively, for patients followed for about 3 years after CTx, while Mogollon [14] reported a prevalence of PTDM of nearly 40% over 5 years. However, the comparison with previous reports is difficult to assess as previous available studies reported observations for a specific follow-up time as opposed to the incidence at 1 year and the prevalence at 3 years. To our knowledge these temporal changes on the rate of PTDM, as well as the description of clinical and para-clinical parameters for those with and without PTDM, have not been reported before in a large cohort of CTx recipients studied over 30 years. Interestingly, data in renal transplant recipients have shown controversial results suggesting that the incidence of PTDM may have increased in the more recent era (before versus after 1995) [17]. In contrast, another study has reported a decrease in the rate of PTDM in the most recent era [18].

The evolution of donor and recipient characteristics from 1983 to 2011 at our institution are in agreement with data reported from the ISHLT registry [15]. As outlined in the registry, we also report an increasing proportion of female recipients, little change in donor gender (approximately 70% males), an increase in donor age, a larger proportion of nonischemic cardiomyopathy, and an increase in the use of mechanical support before CTx. In contrast, we observed a slight increase in recipient age (45±11 in era 1 versus 47±13 in era 3), while the ISHLT registry reported steadily older (median 54 years) recipients since 1992. In contrast with the registry, we reported an increased use of inotropes prior to transplantation. These findings may be related to the small number of VAD used in our cohort. Nevertheless, the increased utilization of inotropes is in agreement with the transplantation of sicker patients in the most recent era.

The ISHLT transplant registry reported no data on the changes in donor weights or in the donor/recipient ratio. However, despite an increase in the rate of obesity in the North American population [19], we reported an increasing weight of donors but no significant changes in recipients BMI over a 30-year follow-up, most likely reflecting strict selection criteria excluding obese patients from the transplant process in our center. The increasing weight ratio found in our study may also be explained by the raising awareness of avoiding undersized donors because this condition has been associated with poorer survival [20].

In this study, we reported novel findings on the long-term changes in other risk factors following CTx such as dyslipidemia. Interestingly, the increase in the rate of dyslipidemia over decades, despite a decreased in mean cholesterol and triglyceride levels, appears counterintuitive. However, these observations may be related to an increase in awareness in the transplant community, as well as changes in the guidelines for the diagnostic and treatment of dyslipidemia published in recent years [21]. Also, a decrease in lipid levels overtime is likely related to the increased use of more powerful statins and other better tolerate drugs such as ezetimibe [22].

In this investigation, we report four independent factors associated with the development of PTDM. Those include tacrolimus immunoprophylaxis, recipient age, duration of cold ischemic time, and use of furosemide. As such, our study adds important and novel observations to the large ISHLT registry that have reported no specific risk factors for PTDM following CTx. Tacrolimus is known to reduce insulin synthesis and secretion within the first months following transplantation [23]. Despite some controversial observations, tacrolimus use has been associated with an increased rate of PTDM after renal transplantation [24, 25]. Nevertheless, this issue has been a matter of controversy following CTx due to the failure of many clinical studies to report a significant impact of tacrolimus on glucose level, HBA1C, and prevalence of diabetes, short-term and long-term, following CTx [26–29]. Our findings suggest that use of tacrolimus immune-prophylaxis may increase the rate of PTDM compared to cyclosporine utilization in an unselected cohort of patients investigated over 30 years following CTx.

The recipients' age, ethnicity, and family history of diabetes are nonmodifiable risk factors for PTDM that have been previously reported in the literature [17, 30]. The majority (98%) of the population studied here was of Caucasian ethnicity, precluding us to screen for an effect of ethnicity. Also, the family history of diabetes was not collected in our database. Although obesity is a well-known risk factor for diabetes, we only computed a trend between recipient body mass index (BMI) and PTDM at 1 year. This may be related to the fact that the proportion of obese patients in our study population was below 10% (7%) for those with a BMI over 30 kg/m^2^ at the time of CTx. Another explanation is that intra-abdominal fat, not assessed here, may be a more significant risk factor than total body weight or BMI [31].

In this study, we also report a significant relationship between the risk of diabetes and cold ischemic time. Although a significant relationship between cold ischemic time and survival has been reported for ischemic times longer than 3 hours [32], no studies have reported the relationship between this specific parameter and the rate of PTDM. The reasons for this are largely unknown. Czer et al. [33] reported an association between longer cold ischemic preservation time and higher rejection score and consequently more advanced graft vascular disease in a rat heterotopic cardiac transplantation model. No human studies have been reported on these findings. Consequently, we speculate that the patients with longer ischemic time may have also presented higher rejection burden leading to an increase in steroid use, which are well known to increase the risk of diabetes. However, we observed no significant differences in the rate of use and or the dose of steroids between patients who developed PTDM compared with those who did not. Other unknown mechanisms are likely involved and require further investigation. Diuretics, specially the thiazide diuretics, are known for their diabetogenic effects. The use and dosage of furosemide have been associated with an increased rate of diabetes following myocardial infarction [34] and in established heart failure, [35] two conditions that often precede CTX. Also the use of furosemide has been associated with beta cell dysfunction [10] and PTDM following renal transplantation [36]. Accordingly, our observations on the association between furosemide use and PTDM following CTX are in agreement with the previous but limited observations reported in the renal transplant population.

Here we reported a significant beneficial impact of MPA on all-cause mortality. The use of MPA has been associated with a decreased rejection rate and better survival rate compared with azathioprine-based therapy following CTx [37, 38]. MPA also impacts microvascular oxygenation and inflammation in an experimental model [39]. In the multivariate model, recipient age was positively associated with mortality, while female donor gender was protective. Previous studies reported that sex mismatch and female donor gender have been associated with adverse outcomes following CTx [40, 41]. However, Kush et al. [42] reported that female recipients of female allografts yielded a 10% decrease in overall mortality. Indeed, in this study female recipients of female allograft exhibited the lowest mortality rate (12 [32%]) compared with the other groups. Nevertheless, the sample size for this specific subgroup was small (*n*=38) and further investigations are needed to better understand this issue.

Previous papers have reported the detrimental effects of PTDM on outcome following renal transplantation [11, 43, 44]. These studies showed that, beyond prognosis, the presence of PTDM decreases graft survival impairs graft function and increases cardiovascular disease and treatment cost. Our observations are in agreement with the data from Mogollon et al. [14] who reported a significant impact of PTDM on renal function long-term following CTX. Our observations are also in agreement with our previous work published by Lachance et al. [6] who reported a significant relationship between some clinical parameters including diabetes and a worsening in renal function post-CTX. There have been some limited observations on the impact of PTDM on outcomes following CTX. These studies have reported an increase in the rate of acute rejection and cardiovascular diseases, but no differences on the short or long-term mortality rate [45–47]. Here we reported that the development of PTDM may increase all-cause mortality. Such differences may be related to the much longer follow-up reported in the present study.

### 4.1. Limitations of the Study

As for any retrospective investigation, some limitations need to be outlined. Laboratory test cut-offs, which we chose to diagnose PTDM, are those recommended by the ADA for the diagnosis of diabetes [48]. Elevated blood glucose levels and the need for hypoglycemic medication are frequent in the early postoperative period [49]. Timing for food intake was not collected in the database. To avoid potential significant bias, patients were classified with PTDM based on the selected parameters screened at the time of discharge and during the outpatient follow-up. Nevertheless, the incidence of PTDM reported here may have been somewhat miss-diagnosed. In fact, oral glucose tolerance tests (OGTT), the gold standard for diagnosing PTDM [50, 51], were not performed routinely in our patients before and after CTx. As such, the rate of PTDM may have been overestimated by missing high-risk patients prior to CTx. On the other hand, HbA1C may be underestimated during the few months following solid organ transplantation, and it is recommended to do an OGTT when HbA1C values are between 5.7 and 6.4% [52]. By choosing a cut-off value of 6.5% from the time of discharge to outpatient follow-up, we may have underestimated the rate of diabetes within the first few months following CTx. However, these patients were likely diagnosed later in the year, when both glucose and HgbA1c had stabilized. Because of these issues we realize that the difference in the rate of PTDM reported here may be difficult to compare with previous studies. More than 95% of our study population were chronically treated by prednisone. As such, the impact of prednisone on PTDM could not be assessed with accuracy using Cox proportional hazard model. Finally, because of the retrospective design of this study, changes in the immunosuppressive medication, the rate of PTDM treated by diet alone, and how many of our patients exhibited transient hyperglycemia remain unknown.

## 5. Conclusions

The rate of PTDM approaches nearly 40% at three years following CTx. This prevalence has not significantly changed over 30 years, despite higher risk for both donors and recipients through the analyzed eras. The development of PTDM is associated with an increase in all-cause mortality and a worsening in renal function over time. Such a high rate of newly diagnosed diabetes justifies aggressive preventive measures very early following CTx in order to minimize long-term complications such as renal dysfunction. Prospective studies using larger sample size are needed to confirm our results and address the proper diagnostic and therapeutic strategies in this high-risk population.

## Figures and Tables

**Table 1:** Characteristics of the transplant population divided by era and according to the development of diabetes at one year.

	Era 1 (CTX 1983-1992) *n *= 104	Era 2 (CTX 1993-2002) *n *= 117	Era 3 (CTX 2003-2011) *n *= 82	Inter-eras Global *p* value	Total *n* = 303	PTDM *n* = 76	No PTDM *n* = 227	PTDM at 1 year *p* value
Follow-up (years)	15.8 (9.7-21.5)	12.3 (9.4-15.7)	5 (3.4-7.8)	<0.0001 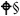	10.6 (5.3-16.4)	8.7(3.6-14.5)	11.8(6.1-16.6)	0.01

Recipient Age (years)	45±11	48±11	47±13	0.0573	47±12	49±11	46±12	0.026

Male gender (%)	90(87)	90(77)	56(68)	0.01 §	236(78)	63(83)	173(76)	0.22

Donor Age (years)	27±9	34±13	37±15	<0.0001 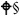	32±13	35±14	31±13	0.063

BMI (kg/m^2^)	23.2 ± 4.6	24.6 ± 4.4	26.2 ± 4.8	0.0014 §¶	25 ± 5	25±3.6	24.7±5	0.51

Weight (kg)	67±13	72±15	76±17	0.0005 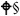	72±15	73±13	71±16	0.38

Donor/Recipient Weight ratio	0.96±0.2	1.05±0.25	1.10±0.25	0.0006 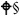	1.04±0.24	1.02±0.21	1.05±0.25	0.37

Gender match	76(73)	76(65)	58(70)		210 (69)	60(79)	150(66)	
Mismatch M->F	5 (5)	13(11)	11(13)	0.1888	29 (10)	3(4)	26(11)	0.06
Mismatch F->M	23(22)	28(24)	13(16)		64 (21)	13(17)	51(22)	

CMP etiology								
Ischemic	63 (61)	52 (44)	27 (33)	0.0007	142(47)	39 (51)	103(45)	0.37
Non ischemic ¥	41 (39)	65 (56)	55 (67)	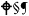	161(53)	37(49)	124(55)	

Pretransplant comorbidities-								

Hyperlipidemia	28 (27)	49 (42)	39 (48)	0.0095 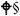	116(38)	35(46)	81(36)	0.11

Cholesterol (mM)	5±1.8	4.2±1.3	3.8±1.2	<0.0001 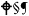	4.3±1.5	4.2±1.6	4.4±1.5	0.48

Triglycerids (mM)	1.4(1.1-1.9)	1.2(0.8-1.6)	1.1(0.8-1.6)	0.0066 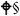	1.3(0.9-1.7)	1.2(0.8-1.8)	1.3(0.9-1.7)	0.71

Inotropic support pre-CTX	35 (34)	45 (38)	42 (51)	0.046 §	122(40)	24(32)	98(43)	0.07

Treatment at discharge								

Diuretics	83(80)	81 (69)	47 (59)	0.0081§	211(70)	58(76)	153(68)	0.17
Furosemide	79(76)	76 (65)	45 (56)	0.0177§	200(66)	57(75)	143(64)	0.068
Thiazide	6 (6)	10 (9)	4 (5)	0.5478	20(6.7)	2(3)	18(8)	0.11
MRA	0	2 (2)	2 (3)	0.3061	4(1.3)	1(1)	3(1)	1

Immuno-prophylaxis				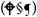				
Cyclosporine	104(100)	109(93)	24(30)	<0.0001	237(79)	48(63)	189(84)	0.0001
Tacrolimus	0	8(7)	54(68)	<0.0001	62(20)	26(34)	36(16)	0.0007
MPA	0	63(54)	78(98)	<0.0001	141(47)	36(47)	105(47)	0.9156
Azathioprine	68(65)	40(34)	0	<0.0001	108(36)	30(39)	78(35)	0.45
Sirolimus	0	1(0.85)	1(1.25)	0.5556	2(0.7)	1(1)	1(0.4)	0.42
Prednisone	104(100)	116(99)	79(99)	0.5556	299(99)	74(97)	225(100)	0.01

CTX, cardiac transplantation; BMI, body mass index; CMP, cardiomyopathy; eGFR, estimated Glomerular Filtration Rate; MRA, mineralo-receptor antagonist; MPA, mycophenolic acid.

Continuous data are presented as mean ± standard deviation or median (lower, upper quartile) if nonparametric and categorical data as counts (percentages).

¥ includes dilated, viral, rheumatic, valvular, noncompaction, hypertrophic, congenital, restrictive, and peripartum cardiomyopathies.

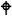
 *p* value < 0.05 for the comparison between era 1 and era 2.

§ *p* value < 0.05 for the comparison between era 1 and era 3.

¶*p* value < 0.05 for the comparison between era 2 and era 3.

**Table 2:** Univariate and multivariate analysis for the risk factors related with the development of PTDM at 1 year.

	Univariate analysis		Multivariate analysis	
Parameters	Hazard Ratio [95% Confidence limits]	*p* value	Hazard Ratio [95% Confidence limits]	*p* value
Donor age	1.017 [1.000, 1.034]	0.0446		

Recipient age (+10 years)	1.27 [1.025, 1.574]	0.0292	1.302 [1.046, 1.621]	0.0182

Recipient BMI	1.060 [1.000, 1.124]	0.0510		

Hyperlipidaemia	1.445 [0.921, 2.269]	0.1094		

Cold ischemic time (+20 min)	1.100 [1.009, 1.199]	0.0303	1.110 [1.012, 1.216]	0.0268

Tacrolimus	2.52 [1.57, 4.05]	<0.0001	3.378 [2.063, 5.530]	<0.0001

Furosemide	1.612 [0.959, 2.710]	0.0714	2.048 [1.185, 3.541]	0.0103

Thiazide diuretics	0.344 [0.084, 1.400]	0.1362		

Year of transplant		0.0711		
1983 to 1992	0.618 [0.357, 1.071]	0.0863		
1993 to 2002	0.549 [0.318, 0.945]	0.0305		
2003 to 2011	- - -	- - -		

Gender mismatch		0.1072		
No mismatch	- - -	- - -		
Female recipient/Male	0.342 [0.107, 1.091]	0.0699		
donor				
Male recipient/Female	0.682 [0.375, 1.243]	0.2116		
donor				

**Table 3:** Multivariate analyses for all cause death and renal failure.

Parameters	*p* value	Hazard Ratio [95% Confidence limits]
**Association with all cause death**	0.0847	2.452 (0.884-6.799)
PTDM		

Recipient age (+10 years)	<0.0001	1.694 [1.353, 2.121]

Donor gender - female	0.0313	0.624 [0.406, 0.959]

MPA no intake	0.0148	1.999 [1.146, 3.489]

**Association with renal failure**		

Recipient age (+10 years)	0.0018	1.432 [1.143, 1.795]

Recipient female gender	0.0048	2.062 [1.247, 3.409]

Furosemide	0.0105	2.019 [1.179, 3.46]

PTDM time-dependent	0.0311	4.882 [1.155, 20.644]

Duration of hospitalization after transplant (+1 day)	0.0021	1.03 [1.011, 1.049]

Renal failure was defined as the occurrence of eGFR < 30ml/min/1.73m^2^.

## Data Availability

The data used to support the findings of this study are available from the corresponding author upon request.

## Abbreviations

ADA: American Diabetes Association
BMI: Body mass index
CTx: Cardiac transplant
CVD: Cardiovascular disease
eGFR: excreted glomerular filtration rate
HBA1C: Glycated hemoglobin
ISHLT: International Society of Heart and Lung Transplant
MPA: Mycophenolate acid
NODAT: New-onset diabetes after transplantation
OGTT: Oral glucose tolerance test
PTDM: Posttransplant diabetes mellitus.
